# Terpenoid biosynthesis in *Arabidopsis* attacked by caterpillars and aphids: effects of aphid density on the attraction of a caterpillar parasitoid

**DOI:** 10.1007/s00442-017-3985-2

**Published:** 2017-10-20

**Authors:** Anneke Kroes, Berhane T. Weldegergis, Francesco Cappai, Marcel Dicke, Joop J. A. van Loon

**Affiliations:** Laboratory of Entomology, Wageningen University, P.O. Box 16, 6700 AA Wageningen, The Netherlands

**Keywords:** *Diadegma semiclausum*, Herbivore-induced plant volatiles, Indirect defence, Multiple attack, Terpene synthase

## Abstract

One of the responses of plants to insect attack is the production of volatile organic compounds that mediate indirect defence of plants by attracting natural enemies of the attacking herbivores. Herbivore-induced plant volatiles (HIPVs) include terpenoids that play key roles in the attraction of natural enemies. Crosstalk between phytohormonal signalling pathways is well known to affect the regulation of plant defences, including the emission of HIPVs. Thus, simultaneous feeding on the same plant by caterpillars and aphids, can affect the attraction of parasitoids by the plant compared to single insect attack. The role of aphid density in the regulation of HIPV emission by plants under dual attack has not been studied previously. Here, we investigated the attraction of *Diadegma semiclausum,* a parasitoid of the Diamondback moth *Plutella xylostella,* to volatiles emitted by *Arabidopsis thaliana* plants, simultaneously attacked by host caterpillars, and by the non-host aphid *Brevicoryne brassicae.* Our study shows that the effect of aphid infestation on parasitoid attraction is influenced by the density of the aphids. Biosynthesis and emission of (*E*,*E*)-α-farnesene could be linked to the observed preference of *D. semiclausum* parasitoids for the HIPV blend emitted by plants dually infested by caterpillars and aphids at a high density compared to dually infested plants with a low aphid density. Parasitoids such as *D. semiclausum* are important enemies of herbivorous insects and a better understanding of how plants express indirect defence mechanisms in response to multiple insect attack will provide important knowledge on plant–herbivore–parasitoid interactions under multiple stress conditions.

**Electronic supplementary material:**

The online version of this article (doi:10.1007/s00442-017-3985-2) contains supplementary material, which is available to authorized users.

## Introduction

When facing an attack by herbivorous insects, plants may respond with structural, molecular and chemical defence mechanisms (Dicke and Van Poecke 2002; Kessler and Baldwin 2002; Schoonhoven et al. 2005; Howe and Jander 2008). One of the chemical responses to insect attack is the production of an array of volatile organic compounds that mediate indirect plant defence by attracting natural enemies of the attacking herbivores (Heil 2008; Dicke and Baldwin 2010). Herbivore-induced plant volatiles (HIPVs) include terpenoids, green leaf volatiles (GLVs) and volatile methyl esters of phytohormones (e.g. methyl salicylate and methyl jasmonate) (Arimura et al. 2005; Mumm and Dicke 2010). The emission of HIPVs may vary depending on the attacking herbivore species, density or developmental stage (Clavijo McCormick et al. 2012; Cai et al. 2013; Pashalidou et al. 2015), and parasitoids and predators use specific blends of HIPVs as cues to locate their herbivore hosts or prey feeding on the plant (De Boer et al. 2004; De Rijk et al. 2013). Therefore, the composition of the HIPV blend plays an important role in the attraction of natural enemies to herbivore-infested plants. For a few herbivore–plant–natural enemy systems particular volatile compounds have been found to affect the attraction of predators and parasitoids to an HIPV blend. For instance, the volatile compounds (*E,E*)-4,8,12-trimethyltrideca-1,3,7,11-tetraene ((*E*,*E*)-TMTT) and methyl salicylate (MeSA) increased the preference of the predatory mite *Phytoseiulus persimilis* for prey-infested lima bean plants (De Boer et al. 2004). Herbivore-induced MeSA decreased the attraction of the parasitoid *Diadegma semiclausum* (Snoeren et al. 2010), whereas the herbivore-inducible terpene alcohol linalool attracts this parasitoid (Houshyani et al. 2013). Other volatile compounds that could play key roles in the attraction of *D. semiclausum*, a parasitoid of the specialist caterpillar *Plutella xylostella* (Ohara et al. 2003), are the tetranor-diterpene (*E*,*E*)-TMTT and the sesquiterpene (*E*,*E*)-α-farnesene. These volatiles are released as part of an HIPV blend emitted by leaves of *Arabidopsis thaliana* in response to *P. xylostella* feeding (Herde et al. 2008; Huang et al. 2010).

Not only feeding by caterpillars, but also other types of herbivory, such as aphid feeding, induce the release of terpenoids (Du et al. 1998; Dicke et al. 2003). A family of terpene synthase (TPS) genes was identified in the genome of *A. thaliana* (Aubourg et al. 2002; Aharoni et al. 2003). Terpenes are grouped based on the number of carbon atoms they contain, such as monoterpenes (C_10_), sesquiterpenes (C_15_) and diterpenes (C_20_) (Gershenzon and Croteau 1991). The monoterpene alcohol linalool was shown to be produced by terpene synthase 10 (TPS10) in *A. thaliana* (Ginglinger et al. 2013). Furthermore, two closely related terpene synthase genes, *TPS02* and *TPS03*, are responsible for the formation of the monoterpene (*E*)-β-ocimene in *A. thaliana* ecotype Wassilewskija and the sesquiterpene (*E*,*E*)-α-farnesene in ecotype Col-0, respectively (Fäldt et al. 2003; Huang et al. 2010). The (*E*,*E*)-geranyllinalool synthase TPS04 regulates an important step in the biosynthesis of the tetranor-diterpene (*E*,*E*)-TMTT in *A. thaliana* (Herde et al. 2008). The extensive knowledge of genes involved in the biosynthesis of HIPV in *A. thaliana,* the availability of well-characterized mutants and knowledge of tritrophic interactions involving this model plant of molecular genetics, makes this plant a suitable object for the present study (van Poecke et al. 2001; van Poecke 2007; Herde et al. 2008, Huang et al. 2010; Tholl and Lee 2011).

The induction of plant volatile biosynthesis is regulated by two main plant defence signalling pathways, the jasmonic acid (JA) and salicylic acid (SA) pathways (Ozawa et al. 2000; Arimura et al. 2005; Pieterse et al. 2012). The JA-signalling pathway regulates the biosynthesis of volatile terpenoids and GLVs (Dicke and Van Poecke 2002), whereas MeSA is synthesized in plants from SA (Chen et al. 2003; Liu et al. 2010). It is well established that leaf-chewing herbivores, such as caterpillars, induce especially JA-mediated defence responses, while phloem-feeding insects, such as aphids, trigger mainly the SA- as well as the JA-signalling pathway (De Vos et al. 2005; Stam et al. 2014). When caterpillars and aphids simultaneously feed on the same plant, a common event in nature, crosstalk between both signalling pathways may affect the regulation of plant defences (Stam et al. 2014). In addition, it is known that phloem-feeding herbivores such as aphids induce lower levels of HIPV emission compared to chewing herbivores (Turlings et al. 1998; Rodriguez-Saona et al. 2003; Ali and Agrawal 2012; Truong et al. 2014). Consequently, multiple herbivores feeding on plants interact indirectly through plant-mediated effects and this may alter the emission of HIPVs (Rodriguez-Saona et al. 2003; Dicke et al. 2009; Ponzio et al. 2013) such that it affects the attraction of predators and parasitoids by the plant, compared to single insect attack (Zhang et al. 2009; Erb et al. 2010; Zhang et al. 2013). For example, herbivory by the phloem-feeding whitefly *Bemisia tabaci* interfered with indirect defences of *A. thaliana* to *P. xylostella* caterpillars. For this interference by *B. tabaci* intact JA- and ethylene signalling was needed (Zhang et al. 2013). In addition, volatiles emitted by lima bean plants simultaneously infested by the whitefly *B. tabaci* and the spider mite *Tetranychus urticae* were less attractive to predatory mites compared to *T. urticae*-infested plants. This effect on the attraction of the predatory mite was the result of a reduction in JA-mediated emission of the monoterpene (*E*)-β-ocimene (Zhang et al. 2009). The same study showed that plant-mediated interference by whiteflies with indirect defence against spider mites was aphid-density dependent (Zhang et al. 2009).

Herbivore density influences the intensity of feeding damage and, consequently, may modulate interactions between plants and multiple insect attacks (Kroes et al. 2015). Therefore, herbivore density may also influence the attractiveness of herbivore-infested plants to parasitoids and predators. However, knowledge about how multiple herbivory influences the composition of volatile blends and, thus, attraction of parasitoids or predators, is limited (Ponzio et al. 2013).

This study addressed the effects of dual herbivory on induced indirect plant defence, by investigating volatile blend composition and expression of volatile-biosynthesis genes of plants attacked by single or multiple herbivores. In addition, effects of differences in HIPV emission on the attraction of parasitoids were assessed by using well-characterized mutant plants. We investigated indirect defence responses of *A. thaliana* wild-type plants and volatile-biosynthesis mutants when dually infested by *P. xylostella* caterpillars and *Brevicoryne brassicae* aphids compared to plants infested by *P. xylostella* caterpillars alone. The plants were infested with either a low or a high aphid density to study aphid-density-dependent effects on plant-mediated interactions between *P. xylostella* and its parasitoid *D. semiclausum*. We assessed the responses of the parasitoid to HIPVs emitted by dually infested plants and by caterpillar-infested plants. To better understand the underlying mechanisms of induced indirect defence to multiple insect attack, the expression profile of genes important for the biosynthesis of plant volatiles and volatile compounds emitted were linked to the behavioural responses of the parasitoid.

## Materials and methods

### Plants and growth conditions

Plants of *Arabidopsis thaliana* ecotype Columbia-0 (Col-0) were used as wild type. Seeds of a mutant defective in the biosynthesis of methyl salicylate, *bsmt1* (*benzoic acid and salicylic acid carboxyl methyltransferase1*; SALK_140496c; Snoeren et al. (2010)) were obtained from the European Arabidopsis Stock Centre (NASC, Nottingham, United Kingdom). Seeds of mutants defective in the biosynthesis of linalool, *tps10* [*terpene synthase10*; Ginglinger et al. (2013)] and of (*E*,*E*)-α-farnesene, *tps03* [*terpene synthase03*; Huang et al. (2010)] were kindly provided by Thierry Delatte (Laboratory of Plant Physiology, Wageningen University, The Netherlands) and Dorothea Tholl (Department of Biological Sciences, Virginia Polytechnic Institute and State University, USA). Seeds of both wild-type and mutant plants were sown in autoclaved (80 °C for 4 h) potting soil (Lentse potgrond, Lent, The Netherlands). After 10–14 days of growth, plants were transferred to individual pots (5 cm diameter) containing soil from the same source. Plants were cultivated in a growth chamber at 21 ± 2 °C under an 8L:16D cycle [200 μmol m^−2^ s^−1^ photosynthetic active radiation (PAR) light intensity] and 60 ± 10% relative humidity (RH). Five- to six-week-old plants were used in the experiments. During the experiments, all plants remained in the vegetative state.

### Insects

Both the cabbage aphid, *B. brassicae* L. (Hemiptera: Aphididae), and the diamondback moth, *P. xylostella* L. (Lepidoptera: Yponomeutidae), were reared on Brussels sprouts plants (*Brassica oleracea* var. *gemmifera* cv Cyrus) at 22 ± 1 °C, 50–70% RH, 16L:8D cycle. The parasitoid *D. semiclausum* Hellén (Hymenoptera: Ichneumonidae) was reared on *P. xylostella* feeding on Brussels sprouts plants at 22 ± 1 °C, 60–70% RH, 16L:8D cycle. Newly emerged wasps were collected and kept in a cage supplemented with 6–10% sugar water solution in a climate cabinet at 21 ± 1 °C with a 16L:8D cycle. In all experiments, female parasitoids were naïve, i.e. without oviposition experience, 3–10 days old and mated.

### Olfactory responses of *Diadegma semiclausum*

Preference of *D. semiclausum* parasitoids was analysed in a dual-choice test performed in a Y-tube olfactometer. The Y-tube olfactometer consisted of two 5-L glass jars which were each connected to one arm of a glass Y-tube. Incoming charcoal-filtered compressed air regulated at a flow of 2 L min^−1^ was led into each of the two glass jars containing an odour source (four *A. thaliana* plants). Prior to placing a plant in one of the jars, the pot of the plant was carefully wrapped in aluminium foil.

At the start of the behavioural assay, a single female parasitoid was released at the base of the Y-tube. Behaviour of the parasitoid was observed in the Y-tube olfactometer for 10 min and its choice for either odour source was recorded when the parasitoid spent at least 15 s beyond a line marked 2 cm from the end of each Y-tube arm. Parasitoids that did not choose within the observation period were excluded from the statistical analysis. After five parasitoids were tested, the position of the odour sources was exchanged to exclude positional bias in the setup. In total four sets of plants and 45–60 parasitoids were tested per combination of odour source. Each set of plants was tested on a different day with a new set of parasitoids. Each parasitoid was only tested once.

As odour source, four *A. thaliana* plants were subjected to one of the following treatments:

1. Uninfested control (undamaged).
2. Infested with two second-instar (L2) *P. xylostella* caterpillars (indicated as ‘*P. xylostella*’ infestations).
3. Simultaneously infested with five adult *B. brassicae* aphids, ‘low density’ (LD), and two *P. xylostella* L2 caterpillars (indicated as ‘dual’ infestations).
4. Simultaneously infested with 25 adult *B. brassicae* aphids, ‘high density’ (HD), and two *P. xylostella* L2 caterpillars (indicated as ‘dual’ infestations).

Insects were allowed to feed freely on the plants. Individual plants were placed in a plastic container (diameter 8 cm × height 14 cm), covered with gauze cloth and closed with elastic bands. Containers were randomly distributed in a tray (12–15 containers per tray). Trays were placed in a growth chamber with a 16L:8D cycle (200 μmol m^−2^ s^−1^ PAR), at 21 ± 2 °C and 50–70% RH. Three days after infestation, plants were used in the behavioural assay. Additionally, after each assay, *P. xylostella* caterpillars were removed from the plants and individually weighed on a microbalance (accuracy 1 μg; CP2P, Sartorius AG, Göttingen, Germany).

The behavioural responses of *D. semiclausum* to HIPV blends from Col-0 plants, *tps03*, *tps10* or *bsmt1* mutant plants were investigated for the following comparisons: (a) *P. xylostella*-infested plants versus undamaged control plants were investigated to assess whether the parasitoid is attracted to volatiles induced by its host, (b) dual LD plants versus Dual HD plants to assess whether the parasitoid discriminates between HIPV emitted from plants with non-hosts at either high or low density. The effect of aphid infestation on the attraction to HIPVs from *P. xylostella*-infested plants was investigated by comparing the response to the HIPV blend of *P. xylostella*-infested Col-0 plants versus either Dual LD or Dual HD Col-0 plants.

### Gene expression analysis

To link behavioural responses of *D. semiclausum* to the transcription of genes important for the biosynthesis of plant volatiles, we additionally performed a gene-expression analysis on Col-0 plants, *tps03, tps10* and *bsmt1* mutants that were used to assess parasitoid preference.

Before tissue collection, insects were removed from the plants with a fine brush. For each treatment, a total of eight leaves from four different plants used as odour source in the Y-tube behavioural assay were pooled to obtain one biological replicate. We selected fully expanded leaves that displayed insect feeding damage. In total, four biological replicates per genotype per treatment were used. Leaf tissue was snap-frozen in liquid nitrogen and stored at − 80 °C prior to analysis.

Finely ground, frozen plant leaf tissue was used for isolation of total RNA with the RNeasy Plant Mini Kit (Qiagen, Hilden, Germany). Total RNA samples were treated with DNase (Qiagen, Hilden, Germany). With the help of the iScript cDNA synthesis Kit (Bio-Rad), cDNA was synthesized from 1 µg RNA. Quantitative RT-PCR analysis was performed in a CFX96 Touch™ Real-Time PCR Detection System (Bio-Rad). Each reaction was performed in a total volume of 25 µL containing 12.5 µL SYBR Green Supermix (Bio-Rad), 5 µL cDNA and 1 µL of 10 µM forward and reverse gene-specific primer pair. For each reaction, two technical replicates were performed and average values were used in the analyses. The studied genes were the terpene synthase (TPS) genes *TPS03* (At4g16740), *TPS04* (At1g61120) and *TPS10* (At2g24210), the salicylic acid methyl transferase gene *BSMT1* (At3g11480) and the two reference genes *ELONGATION FACTOR 1α* (*EF1α*) (At5g60390) and *GLYCERALDEHYDE*-*3*-*PHOSPHATE DEHYDROGENASE* (*GAPDH*) (At3g04120). The following thermal profile was used for reactions with *TPS03*, *TPS04* and *BSMT1*: 3 min 95 °C, followed by 40 cycles of 15 s at 95 °C, and 45 s at 60 °C. For reactions with *TPS10* thermal conditions consisted of 3 min 95 °C, followed by 40 cycles of 15 s at 95 °C, and 45 s at 62 °C.

The two reference genes, *GAPDH* and *EF1α*, were carefully selected after evaluating their expression stability by calculating the geNorm value and coefficient of variation (CV) (qbase+ v. 2.6.1, Biogazelle; Hellemans et al. 2007). Relative expression for each tested gene was calculated by using the geometric mean of threshold cycle (Ct) values (Vandesompele et al. 2002) from the two reference genes with the 2^−ΔΔCt^ method (Livak and Schmittgen 2001).

### Headspace collection

Plant volatiles were collected from four *A. thaliana* Col-0 plants subjected to one of the four treatments as described in the ‘Olfactory responses of *D. semiclausum*’ section above. We used Col-0 plants as a marker to identify plant volatile profiles induced by multiple insect attacks. Future studies should explore the use of volatile-biosynthesis mutants for plant volatile collection to gain additional insight into plant responses to multiple herbivory.

For each treatment, seven replicates were sampled. The pots containing the four plants were carefully wrapped in aluminium foil and placed in a clean 5-L glass jar. The jars were sealed with a Viton-lined glass lid with an air inlet and outlet. Volatile control samples were collected from empty glass jars and from aluminium-wrapped pots filled with soil in order to correct for non-plant-related volatiles. Prior to volatile collection, the jars were ventilated for 30 min using charcoal-filtered compressed air. Plant volatiles were collected on 200 mg Tenax TA (20/35 mesh; CAMSCO, Houston, TX, USA) in a stainless steel cartridge by drawing air from the jars using an external pump at 200 mL min^−1^ for 6 h, starting at 10 am. Immediately after each volatile collection, insects were removed from the plants and plant shoots of each treatment were pooled and weighed on an analytical balance (accuracy 0.1 mg; Mettler Toledo ML54/01). The Tenax TA cartridges were dry-purged under a stream of nitrogen (N_2_, 50 mL min^−1^) for 10 min and stored at room temperature (22 ± 2 °C) until analysis.

### Chemical analysis of volatiles

Plant volatiles were identified and quantified as described by Pangesti et al. (2015). Separation and detection of plant volatiles was done using a Thermo Trace Ultra gas chromatograph (GC) coupled to a Thermo Trace DSQ quadrupole mass spectrometer (MS) (Thermo Fisher Scientific, Waltham, USA). The volatiles were thermally released from the Tenax TA cartridges at 250 °C for 10 min with a helium flow of 20 mL min^−1^ on an Ultra 50:50 thermal desorption unit (Markes, Llantrisant, UK), while being focused on a cold sorbent trap at 0 °C (Unity, Markes). After completion of the desorption process, volatile compounds were released from the cold trap by ballistic heating at 40 °C s^−1^ to 280 °C, which was maintained for 10 min and were then transferred in a splitless mode to an analytical column [(ZB-5MSi; 30 m × 0.25 mm i.d. × 0.25 µm film thickness with 5 m built-in guard column (Phenomenex, Torrance, CA, USA)] situated inside the GC oven. The temperature of the GC oven was initially held at 40 °C for 2 min, which was then raised at 10 °C min^−1^ to a final temperature of 280 °C and held for 4 min under a helium flow of 1 mL min^−1^. The DSQ MS was operated in a scan mode with 35–350 amu mass range at 5.38 scans s^−1^ and spectra were recorded in electron impact ionization (EI) mode at 70 eV. MS transfer line and ion source were set to 275 and 250 °C, respectively. Volatile compounds were tentatively identified by comparison of mass spectra with those in the NIST 2005 and the Wageningen Mass Spectral Database of Natural Products MS libraries, as well as using experimentally obtained linear retention indices (LRI).

### Statistical analyses

To determine whether parasitoid preferences and response rates differed between the various odour sources, data on olfactory responses of *D. semiclausum* were analysed using a *χ* ^2^ test in SPSS v. 22.0 (SPSS Inc., Chicago, IL, USA) for each choice situation tested. In addition, data were analysed using a generalized linear model (GLM) with Poisson distribution and log link function in GenStat v. 17 (VSN International, Hemel Hempstead, UK) to compare choice distributions between plant genotypes. Genotype and treatment combination (i.e. undamaged plants tested in the Y-tube olfactometer against *P. xylostella*-damaged plants or plants infested by both *P. xylostella* and a low density of five aphids per plant (hereafter abbreviated as Dual LD for dual low density) tested against plants infested by both *P. xylostella* and a high density of 25 aphids per plant [abbreviated as Dual HD for dual high density)] and the interaction genotype × treatment combination were included as fixed factors for data on proportion of responsive or non-responsive wasps. In the choice assays involving undamaged plants, the number of wasps choosing the *P. xylostella*-infested plants out of the total number of responding wasps was entered as the response variable. In the choice assays between Dual LD versus Dual HD, the number of wasps choosing the Dual HD plants out of the total number of responding wasps was entered as the response variable. The dispersion parameter was estimated to account for residual variance. Post hoc comparisons for proportion of responsive or non-responsive wasps were analysed with a least significant difference (LSD) test.

Fisher’s exact test (two-tailed) was used to determine whether parasitoid preferences were distributed identically across different days on which the tests were repeated. After each behavioural bioassay, we tested if there were differences in weight of *P. xylostella* caterpillars feeding alone or simultaneously with aphids at low or high density on plants. Data of *P. xylostella* larval weight were analysed with a linear mixed model with treatment as fixed factor and experimental group (i.e. the four *A. thaliana* plants subjected to one of treatments used in the behavioural bioassay) as random factor. Effect of treatment on plant shoot fresh weight was analysed with an independent samples *t* test. The statistical analysis of *P. xylostella* larval weight and plant shoot weight was carried out using SPSS v. 22.0 (SPSS Inc., Chicago, IL, USA).

The expression of genes and the quantity of each volatile emitted by plants on which caterpillars were feeding alone or simultaneously with aphids at either density or left undamaged were compared using a GLM with Poisson distribution and log link function in GenStat v. 17.0 (VSN International, Hemel Hempstead, UK). The factor treatment was included in the model as fixed factor. The dispersion parameter was estimated to account for residual variance. Post hoc comparisons for gene expression and volatile data were analysed with an LSD test. Data on volatile emission were also investigated by discriminant analysis. The quantified peak areas of individual volatile compounds were divided by plant shoot fresh mass, log-transformed, univariate scaled and mean-centred prior to subjecting the data to a multivariate data analysis: orthogonal projection to latent structures discriminant analysis (OPLS-DA) using SIMCA-P+ version 14.0 statistical software (Umetrics AB, Umeå, Sweden). The analysis determines whether samples from different treatment groups can be separated on the basis of quantitative and qualitative differences in their volatile blends. The results of the analysis are visualized in score and loading plots. The score plot identifies patterns that discriminate between the sample groups according to the two given model components of OPLS-DA, i.e. the predictive and orthogonal component. The predictive component corresponds to variation between the sample treatments, whereas the orthogonal component corresponds to within-sample variation. The loading plot displays the contribution and variable importance in the projection (VIP) of each volatile compound for the discrimination between the sample groups. Volatile compounds with VIP > 1 are considered most influential in the model (Eriksson et al. 2013). Pair-wise OPLS-DA analyses were conducted on the volatile blends of the different treatment groups. The quality of each OPLS-DA model was evaluated using the parameter *R* ^2^ *X*, which is used to assess the stability of the model (providing a quantitative measure of the explained variation) and indicates goodness of fit (Eriksson et al. 2013).

## Results

### Olfactory responses of the caterpillar parasitoid *Diadegma semiclausum* to HIPV blends

**Fig. 1:**
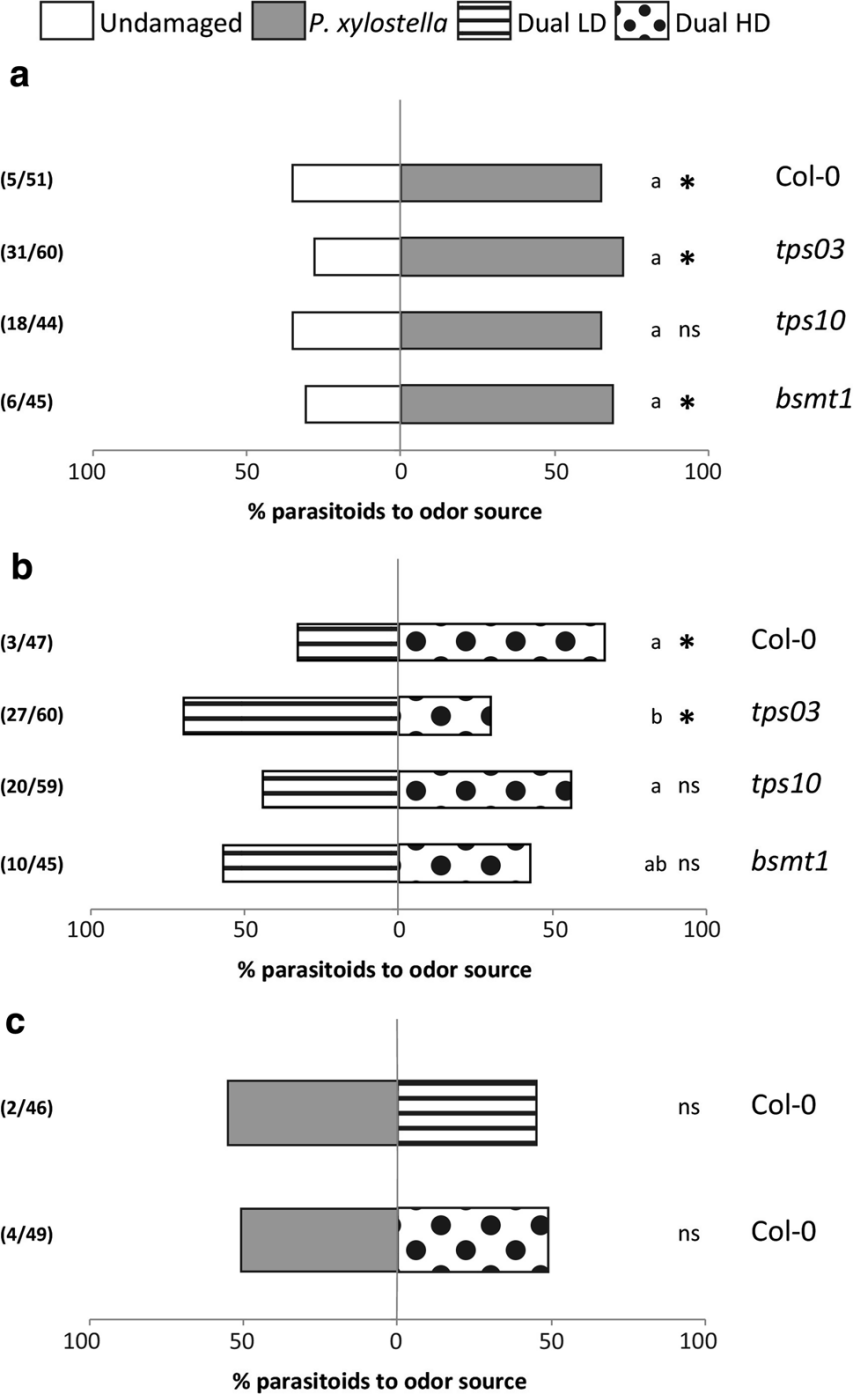
Preference of *D. semiclausum* in a Y-tube olfactometer to volatile blends emitted by *Arabidopsis* Col-0 wild type, *tps03*, *tps10* or *bsmt1* mutants after three days of insect infestation. Undamaged plants were tested against plants infested by two L2 *P. xylostella* caterpillars (**a**), plants dually infested by caterpillars and a low density of five *B. brassicae* aphids (Dual LD) were tested against plants dually infested by caterpillars and a high density of 25 *B. brassicae* aphids (Dual HD) (**b**), or Col-0 plants infested by caterpillars were tested against Col-0 plants dually infested by *P. xylostella* caterpillars and a low aphid density (**c**), or Col-0 plants infested by caterpillars were tested against plants dually infested by *P. xylostella* caterpillars and aphids at a high density (**c**). Each bar represents the percentage of wasps choosing for each of the two odour sources, which consisted of four plants per treatment. For each pair-wise comparison, 3–4 sets of plants were tested on different days. An asterisk indicates a significant preference within a dual-choice test: *ns* not significant; asterisk, *P* < 0.05 (*χ* ^2^ test). Parasitoid preference that is significantly different between the different genotypes is indicated with different letters (GLM, *P* < 0.05). Numbers in parentheses represent number of non-responsive wasps and total number of tested wasps, respectively

Parasitoid preference was studied for caterpillar-infested versus uninfested Col-0 plants and mutants impaired in the biosynthesis of linalool (synthesized by TPS10), MeSA (synthesized by BSMT1) and (*E*,*E*)-α-farnesene (synthesized by TPS03). Female *D. semiclausum* parasitoids preferred volatiles emitted by *P. xylostella*-infested plants of Col-0 wild type, as well as *tps03* and *bsmt1* mutants over those from undamaged Col-0, *tps03* or *bsmt1* plants (Fig. 1a; *χ* ^2^ test, Col-0: *χ* ^2^ = 4.261, *P* = 0.039; *tps03: χ* ^2^ = 5.828, *P* = 0.016; *bsmt1*: *χ* ^2^ = 5.769, *P* = 0.016). Parasitoids did not discriminate between *P. xylostella*-infested *tps10* mutants and undamaged *tps10* mutants (Fig. 1a; *χ* ^2^ test, *χ* ^2^ = 2.462, *P* = 0.12).

When comparing parasitoid responses to dually infested plants with either a low or high aphid density, the parasitoids discriminated between volatiles from Dual LD and Dual HD plants for the Col-0 plants and *tps03* mutant. The parasitoids preferred the volatile blend from Dual HD over volatiles emitted by Dual LD plants for Col-0 (Fig. 1b; *χ* ^2^ test, *χ* ^2^ = 5.233, *P* = 0.022), whereas the opposite was recorded for *tps03* plants: parasitoids significantly preferred the volatile blend from Dual LD *tps03* mutants over those from Dual HD *tps03* mutants (Fig. 1b; *χ* ^2^ test, *χ* ^2^ = 5.121, *P* = 0.024). The wasps did not discriminate between the volatile blend from Dual LD or Dual HD *tps10* and *bsmt1* mutants (Fig. 1b; *χ* ^2^ test, *tps10*; *χ* ^2^ = 0.641, *P* = 0.42; *bsmt1*: *χ* ^2^ = 0.714, *P* = 0.40).

The presence of *B. brassicae* at low or high density did not interfere with *D. semiclausum*’s response to volatiles from *P. xylostella*-infested plants: parasitoids did not discriminate between the volatile blend from Col-0 plants infested by caterpillars and the volatile blend from Dual LD or Dual HD Col-0 plants (Fig. 1c; *χ* ^2^ test, Dual LD: *χ* ^2^ = 0.364, *P* = 0.55; Dual HD: *χ* ^2^ = 0.022, *P* = 0.88).

To further investigate the role of linalool, MeSA and (*E*,*E*)-α-farnesene in mediating parasitoid preference and responsiveness, we compared parasitoid behaviour in response to herbivore-infested Col-0 plants to those in response to herbivore-infested volatile-biosynthesis mutants. Parasitoid preference was influenced by the different genotypes and treatment combinations tested (Online Resource 6; GLM). There were no significant differences between the different genotypes for the proportion of parasitoids that preferred volatiles from *P. xylostella*-infested plants over those from uninfested plants (Fig. 1a). Interestingly, volatiles emitted by Dual HD *tps03* mutants significantly affected parasitoid preference compared to Dual HD Col-0 plants (Fig. 1b). There were no significant differences for parasitoid preference in response to Dual LD and Dual HD Col-0 plants and to the same treatment combination for *tps10* and *bsmt1* mutants (Fig. 1b).

Analysis of the response of wasps towards volatile blends emitted by either Col-0 plants or volatile-biosynthesis mutants, showed an effect of plant genotype but no effect of the treatment combinations offered (i.e. undamaged versus *P. xylostella* or Dual LD versus Dual HD) (Online Resource 7; GLM). Volatiles emitted by *tps10*, *tps03* and *bsmt1* mutants increased the percentage of wasps that did not make a choice compared to Col-0 plants.

Thus, blocking the biosynthesis of linalool, (*E*,*E*)-α-farnesene and MeSA, does not influence preference of *D. semiclausum* parasitoids for plants infested by *P. xylostella* caterpillars versus uninfested plants. On the other hand, mutations in *TPS03, TPS10* and *BSMT1* reduce the responsiveness of the wasps. In addition, (*E*,*E*)-α-farnesene is required for the density-dependent effect on attraction of parasitoids to plants infested by both caterpillars and aphids.

Preference of *D. semiclausum* parasitoids was not influenced by the day on which the experiments were performed for Col-0 plants and *tps10*, *bsmt1* or *tps03* mutants (*Fisher’s exact test*, *P* > 0.05). Furthermore, caterpillar body mass reached similar values when feeding on Col-0 plants, *tps10*, *bsmt1* or *tps03* mutants (Online Resource 1; LMM, *P* > 0.1) tested during the Y-tube olfactometer bioassays.

### Plant volatile emission

Emission of volatiles was analysed to study if differences in volatile profile between plant treatments could explain the observed differences in parasitoid preference.

**Fig. 2:**
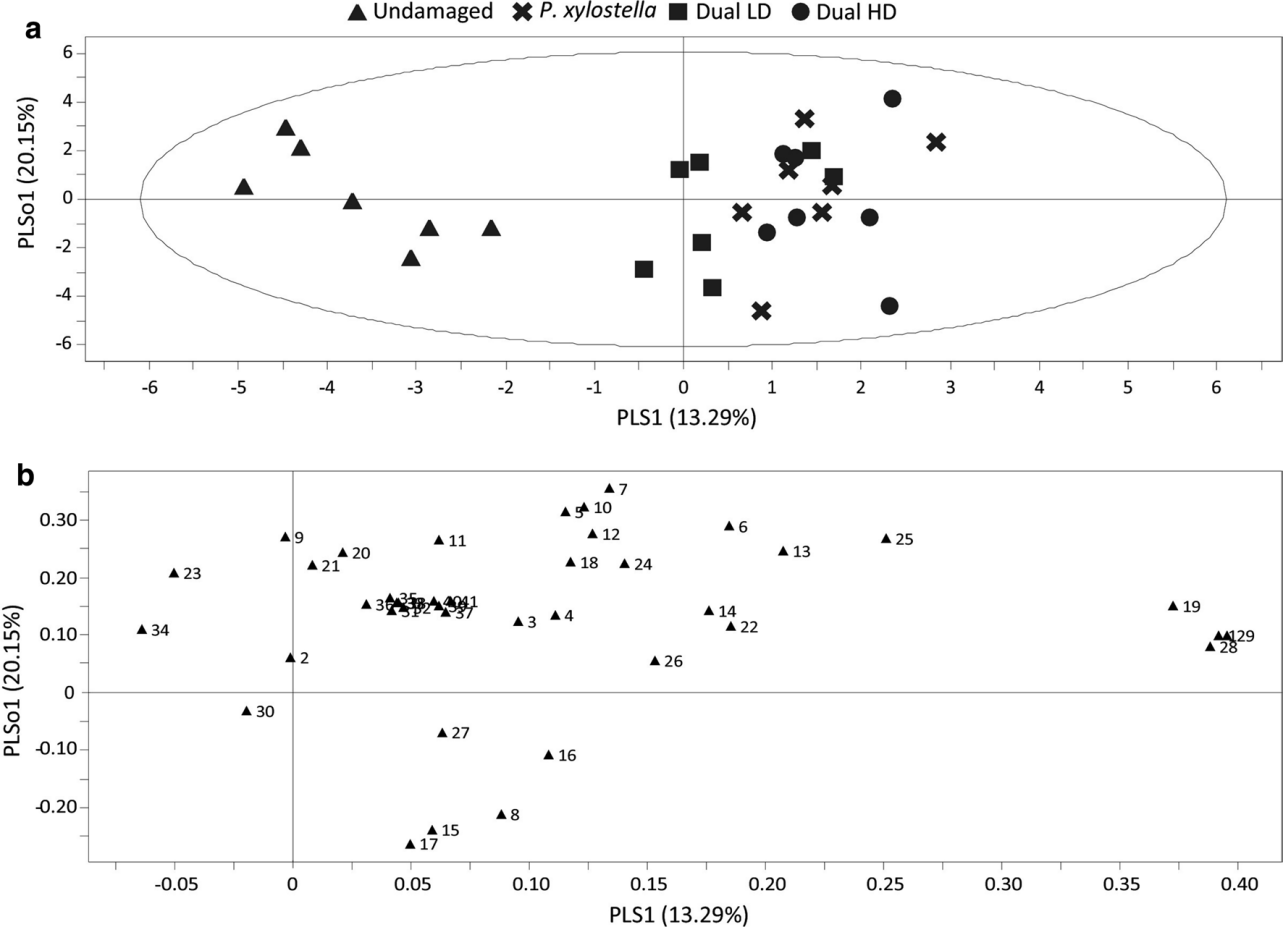
Orthogonal projection to latent structures discriminant analysis (OPLS-DA) of volatile compounds emitted by *Arabidopsis* wild-type Col-0 plants after 3 days of insect infestation. Plants were infested by *P. xylostella* alone, dually infested by *P. xylostella* and *B. brassicae* (Dual) or left undamaged. Plants were infested with either a low (LD) or a high (HD) density of *B. brassicae* aphids. **a** Score plot displaying grouping pattern according to the first two model components and the Hotelling’s ellipse of the 95% confidence interval for the observations. Each point represents one sample (*n* = 7 replicates). The OPLS-DA resulted in a model with one significant predictive and two significant orthogonal components with *R* ^2^ *X* = 0.639. **b** Loading plot of the first two components of OPLS-DA, showing the contribution of each volatile compound to the separation of the four treatments. Numbers refer to the volatile compounds listed in Online Resource 4

In total, 41 different volatile compounds were detected in the headspace of all treatments (Online Resource 4). An OPLS-DA model comparing headspace samples from all four treatments showed differences in volatile blends based on the presence or absence of herbivores. The first two components of the OPLS-DA, i.e. the predictive and orthogonal component, are plotted in the model (Fig. 2a). The predictive component explained 13.29% of the variance, while 20.15% was explained by the first of two orthogonal components. A group of eight compounds contributed most strongly to the model (VIP > 1), indicating that these compounds contributed most to the difference between the volatile blends (Online Resource 5). Based on the three highest VIP-values, 1-penten-3-ol, (*E*,*E)*-TMTT and (*E*,*E*)-α-farnesene influenced the separation of undamaged and herbivore-infested plants the most (Fig. 2b). These three compounds were emitted in significantly higher amounts by herbivore-infested plants than by undamaged plants (Online Resource 4).

**Fig. 3:**
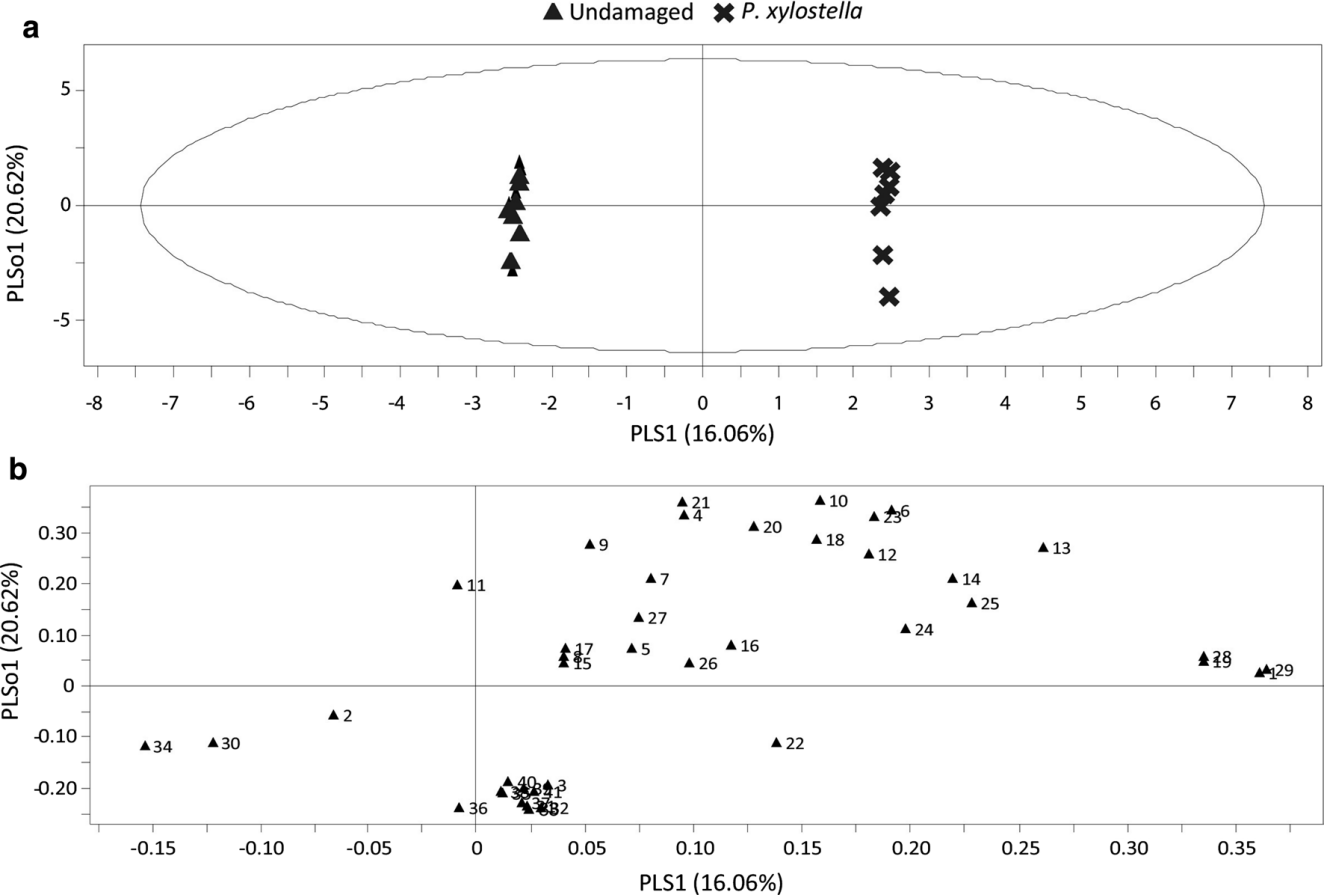
Orthogonal projection to latent structures discriminant analysis (OPLS-DA) of volatile compounds emitted by *Arabidopsis* wild-type Col-0 plants after 3 days of insect infestation. Plants were infested by *P. xylostella* caterpillars or left undamaged. **a** Score plot displaying grouping pattern of samples according to the first two model components and the Hotelling’s ellipse of the 95% confidence interval for the observations. Each point represents one sample (*n* = 7 replicates). The OPLS-DA resulted in a model with one significant predictive and seven significant orthogonal components with *R* ^2^ *X* = 0.908. **b** Loading plot of the first two components of OPLS-DA, showing contribution of each volatile compound to the separation of the two treatments. Numbers refer to the volatile compounds listed in Online Resource 4

Pair-wise comparison by OPLS-DA for volatiles emitted by undamaged plants and plants infested by *P. xylostella* caterpillars shows a clear separation based on the presence or absence of *P. xylostella* caterpillars. The first two components of the OPLS-DA are plotted in the model (Fig. 3a). The predictive component explained 16.06% of the variability, while 20.62% was explained by the first of seven orthogonal components. A group of 12 plant volatile compounds contributed most strongly to the model (VIP > 1), indicating that these compounds contributed to the difference between the volatile blends (Online Resource 5). Based on the four highest VIP-values, (*E*,*E)*-TMTT, 1-penten-3-ol, (*E*,*E*)-α-farnesene and MeSA influenced the separation of volatile blends from undamaged and caterpillar-infested plants the most (Fig. 3b). These four compounds were emitted in significantly higher amounts by caterpillar-infested plants than by undamaged plants (Online Resource 4).

**Fig. 4:**
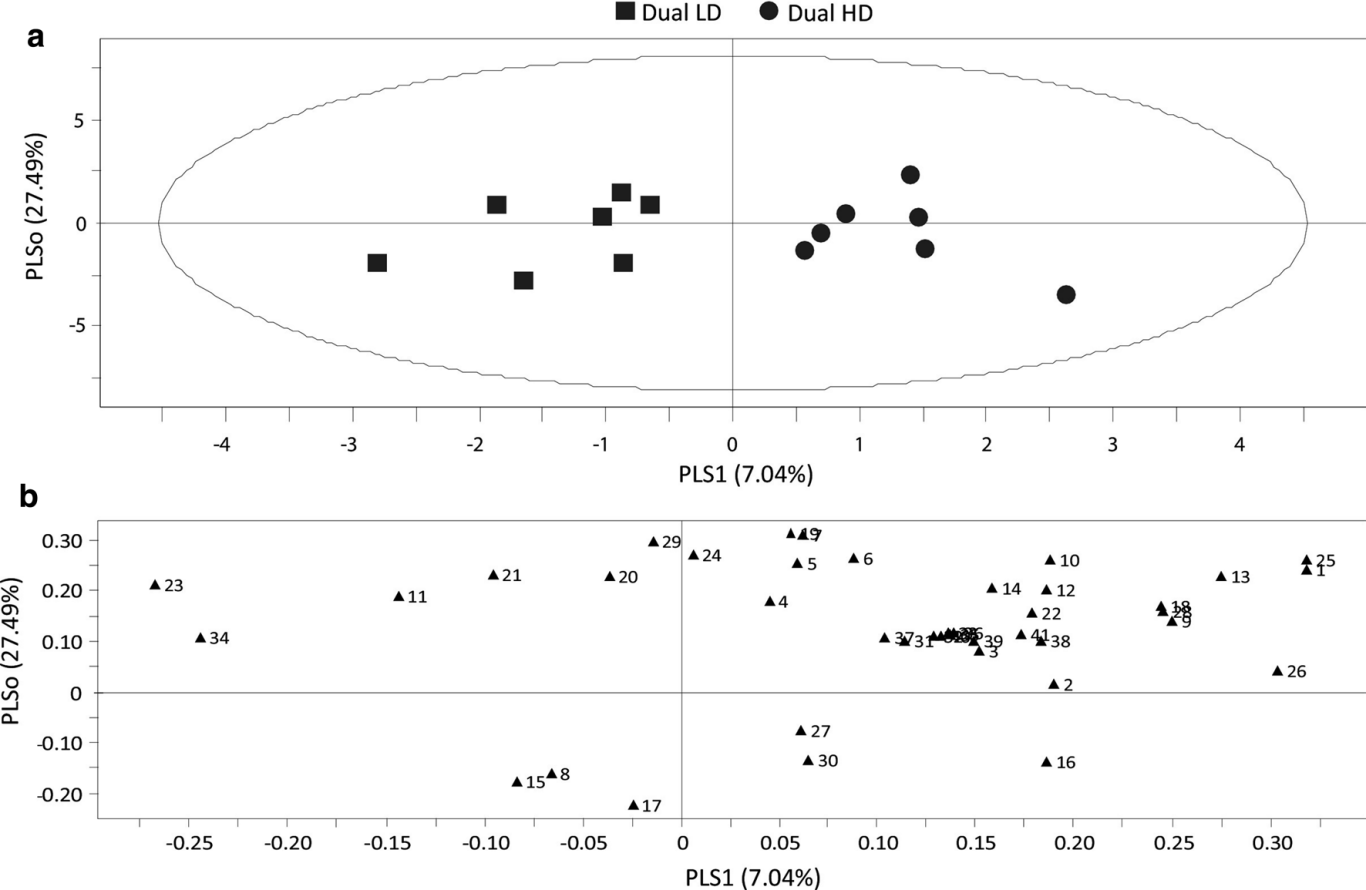
Orthogonal projection to latent structures discriminant analysis (OPLS-DA) of volatile compounds emitted by *Arabidopsis* wild-type Col-0 plants after 3 days of insect infestation. Plants were dually infested by *P. xylostella* and a low *B. brassicae* density (Dual LD, 5 aphids) or by *P. xylostella* and a high *B. brassicae* density (Dual HD, 25 aphids). **a** Score plot displaying grouping pattern according to the first two model components and the Hotelling’s ellipse of the 95% confidence interval for the observations. Each point represents one sample (*n* = 7 replicates). The OPLS-DA resulted in a model with one significant predictive and two significant orthogonal components with *R* ^2^ *X* = 0.620. **b** Loading plot of the first two components of OPLS-DA, showing contribution of each volatile compound to the separation of the two treatments. Numbers refer to the volatile compounds listed in Online Resource 4

An OPLS-DA model including volatiles emitted by Dual LD plants and Dual HD plants showed a clear separation between the two treatments. The first two components of the OPLS-DA model are plotted in the model (Fig. 4a) and explain 34.53% of the total variance. A group of 13 plant volatile compounds contributed most strongly to the model (VIP > 1) (Online Resource 5). Based on the three highest VIP-values, 1-penten-3-ol, (*E*,*E*)-α-farnesene and linalool influenced the separation of the two treatments the most (Fig. 4b).

Volatile blends emitted by *P. xylostella*-infested plants and Dual LD or Dual HD plants were not separated by OPLS-DA.

After each collection of volatile compounds plant shoot fresh weight was measured. There was no effect of insect infestation on plant biomass (Online Resource 2; ANOVA, *P* > 0.1).

### Transcriptional analysis of TPS03, TPS04, TPS10 and BMST1

To explain the observed HIPV-profiles, transcript levels of genes important for their biosynthesis (i.e. *TPS03*, *TPS04* and *TPS10* in the terpenoid biosynthesis pathway, and *BSMT1* in the methyl salicylate biosynthesis pathway) were analysed in Col-0 plants and *tps10, bsmt1* and *tps03* mutants used in the Y-tube behavioural bioassays.

Expression of *TPS03*, *TPS10* and *BSMT1* was verified in the mutants *tps03*, *tps10* and *bsmt1*, respectively. Caterpillar-induced expression of *TPS03*, *TPS10* and *BSMT1* was severely reduced in their corresponding mutants when compared with Col-0 wild-type plants (Online Resource 3; GLM, *P* < 0.02).

**Fig. 5:**
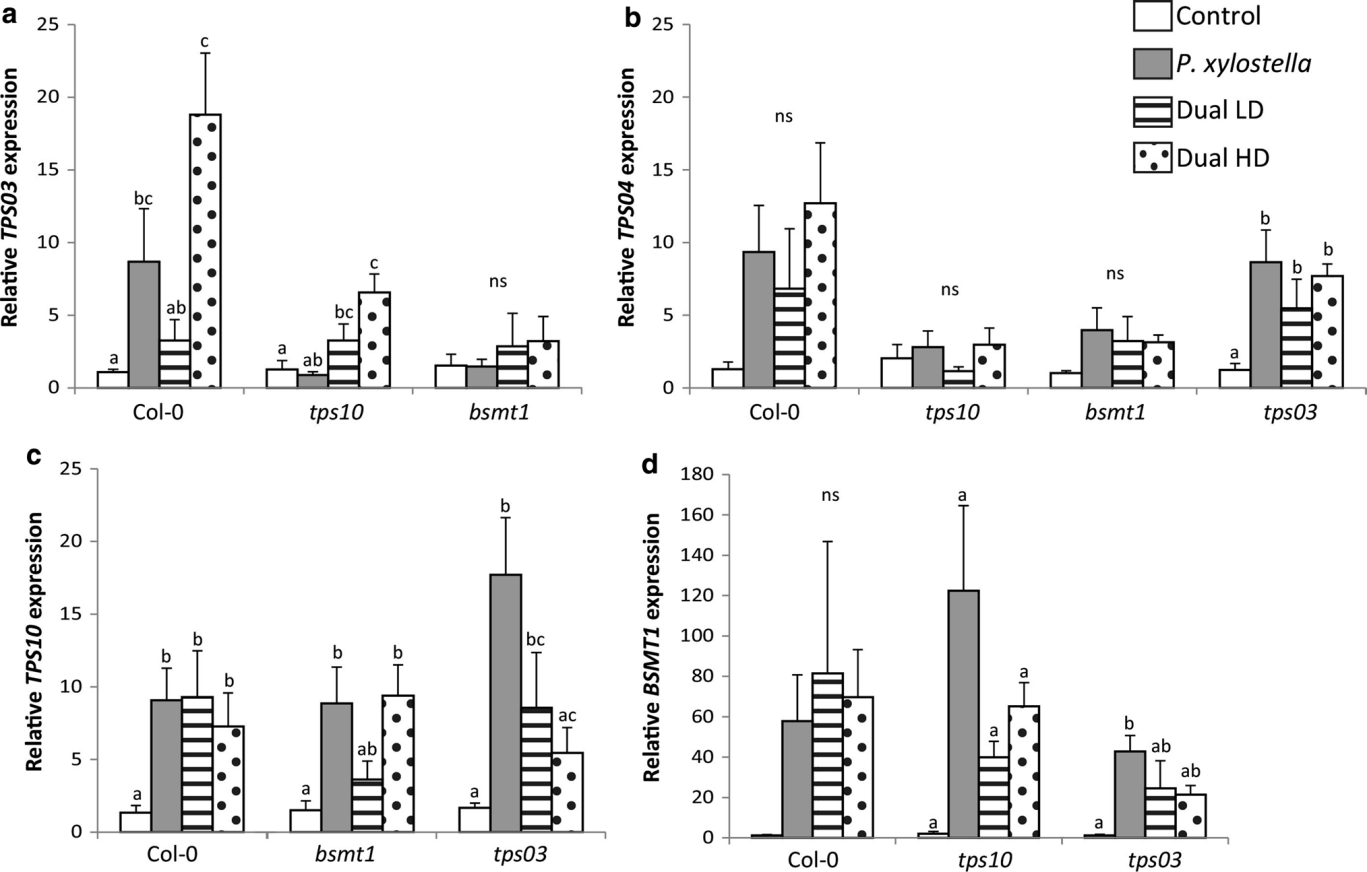
Gene expression in leaves of *A. thaliana* wild-type Col-0 and mutants *tps10*, *bsmt1* and *tps03* used during the Y-tube olfactometer bioassays after single *P. xylostella* and dual *P. xylostella* and *B. brassicae* infestation (Dual) at either a low (LD, 5 aphids) or high (HD, 25 aphids) aphid density and without infestation (control). Bars represent mean ± SE (*n* = 4 biological replications). Bars marked with different letters are significantly different between treatments (GLM, *P* < 0.05; *ns* not significant)

There was a significant effect of treatment on the expression of *TPS03* and *TPS10* in Col-0 plants, of *TPS03* and *BSMT1* in *tps10* mutants, of *TPS10* in *bsmt1* mutants and of *TPS04*, *TPS10* and *BSMT1* in *tps03* mutants (Online Resource 8). However, due to variation in *BSMT1* expression level within treatment type, no significant differences between treatments for *BSMT1* expression level were found for Col-0 plants and *tps10* mutants (Fig. 5).

In Col-0 plants, feeding by caterpillars plus aphids at a high density induced *TPS03* expression to a higher level compared to simultaneous feeding of caterpillars and aphids at low density (Fig. 5a).

This shows that aphids influence *TPS03* expression level in a density-dependent manner. Interestingly, no significant difference was found between *TPS03* expression levels in *tps10* and *bsmt1* mutants infested by caterpillars plus a low or high aphid density (Fig. 5a). Furthermore, expression levels of *TPS03* in *P. xylostella*-infested *tps10* and *bsmt1* mutants remained unchanged compared to uninfested (Control) plants (Fig. 5a).

Feeding by *P. xylostella* caterpillars, regardless of whether aphids were present as well, induced the expression of genes important for the biosynthesis of volatiles in plants. Significantly higher expression levels of *TPS04* in *tps03* mutants, of *TPS10* in Col-0 and *bsmt1* plants, and of *BSMT1* in *tps03* mutants were found upon caterpillar feeding compared to control plants (Fig. 5).

In *tps03* mutants, caterpillars feeding alone and simultaneous feeding by caterpillars and aphids at low density induced *TPS10* expression to a significantly higher level compared to uninfested plants (Fig. 5). *TPS10* expression was negatively correlated with aphid density on caterpillar-infested *tps03* plants (Fig. 5). Expression of *TPS10* is significantly affected by feeding of caterpillars in combination with aphids at high density in *tps03* mutants compared to control plants. Caterpillars and aphids at high density feeding on *tps03* mutants induced significantly lower levels of *TPS10* expression compared to caterpillars feeding alone, which was not found for Col-0 plants (Fig. 5). This indicates that aphids at high density feeding simultaneously with caterpillars interfere with caterpillar-induced *TPS10* expression.

## Discussion

We investigated the effect of simultaneous feeding by *B. brassicae* aphids and *P. xylostella* caterpillars on induced plant responses and the attraction of the parasitoid *D. semiclausum*, an important natural enemy of *P. xylostella* caterpillars. Our study shows that *D. semiclausum* parasitoid attraction is influenced by the density of the aphids. Parasitoids preferred the volatile blend of dually infested plants at high aphid density over those from dually infested plants at low aphid density. No discrimination was recorded when host-infested plants were offered versus host-infested plants with either a low or a high aphid density. This suggests that different aphid densities have opposing effects, which is supported by our previous work on the effects of high and low aphid densities on *P. xylostella* (Kroes et al. 2015).

It has been observed before that the level of induced indirect plant defence is influenced by the density of the herbivores feeding on the plant (Dudareva et al. 2006). For instance, attraction of the predatory mite *P. persimilis* to volatiles from lima bean plants (*Phaseolus lunatus*) infested by spider mites (*T. urticae*) and attraction of the parasitoid *Cotesia vestalis* to volatile blends emitted by *P. xylostella*-infested cabbage plants (*B. oleracea*) are positively density-dependent (Gols et al. 2003; Girling et al. 2011).

Compared with feeding by only one insect species, herbivory by a second herbivore may influence indirect defence responses (Rodriguez-Saona et al. 2003, 2005; Heil 2008; Dicke et al. 2009; Erb et al. 2010; Zhang et al. 2013; Ponzio et al. 2016), which can also be affected by the density of the attacking insects (Zhang et al. 2009; Kroes et al. 2015). Simultaneous feeding by phloem-feeding whiteflies and *Spodoptera exigua* caterpillars on cotton plants (*Gossypium hirsutum*) reduced the emission of DMNT [(*E*)-4,-8-dimethyl-1,3,7-nonatriene], TMTT and the monoterpene myrcene compared to plants infested by only *S. exigua* (Rodriguez-Saona et al. 2003). Zhang et al. (2013) showed that feeding by *B. tabaci* whiteflies significantly reduced the attraction of *D. semiclausum* parasitoids to volatile blends from *A. thaliana* plants simultaneously infested by *P. xylostella*, which was associated with differences in the HIPV blend. Our present results show that feeding by *B. brassicae* aphids does not affect the preference of *D. semiclausum* for *P. xylostella*-infested plants over control uninfested plants. This indicates that aphids and whiteflies, although both phloem feeders, differentially induce plant responses. Our data indicate that feeding by caterpillars plus aphids at a low density suppressed transcription of *TPS03* (encoding an (*E*,*E*)-α-farnesene synthase) compared to simultaneous feeding by caterpillars and aphids at a high density. This indicates that *TPS03* expression in response to both caterpillar and aphid feeding depends on aphid density. In addition, olfactory responses of *D. semiclausum* to volatiles emitted by dual-infested *tps03* mutants confirmed that a functional *TPS03* in *A. thaliana* is required for interference by aphids.

Results from the volatile analysis show that the volatile blends changed depending on simultaneous feeding by caterpillars and aphids at low or high density and this may explain the behavioural responses by *D. semiclausum*. Whether the responses benefit the parasitoids in terms of offspring performance remains to be investigated. Such performance effects may be present in terms of, e.g. offspring development rate and level of host immunity (Rodriguez-Saona et al. 2005; Bukovinszky et al. 2009; Soler et al. 2012).

In the headspace of plants infested by caterpillars alone, dually infested by caterpillars and aphids at both densities or undamaged plants, the same compounds were detected. Moreover, four of these compounds (1-penten-3-ol, (*E*,*E)*-TMTT, (*E*,*E*)-α-farnesene and linalool) were found to be important for the separation of the different blends in the multivariate data analysis. Thus, this study underlines the significance of the quantitative composition of volatile blends used by parasitoids to locate host-infested plants. However, it is noteworthy that parasitoids are able to detect very subtle differences in volatile blends which are difficult to identify by chemical analysis (Clavijo McCormick et al. 2014; Ponzio et al. 2016), leaving the possibility open that other HIPVs contributed to the discrimination exhibited by the parasitoids. In addition, no difference in (*E*,*E*)-α-farnesene emission by dual-infested plants at low density and high density were found, whereas transcript levels of the corresponding *TPS03* gene did differ between treatments. This may be related to substrate availability (Degenhardt et al. 2009; Tholl and Lee 2011) or through posttranslational protein modifications (Tholl et al. 2005). Furthermore, sesquiterpenes, such as (*E*,*E*)-α-farnesene, are known to be unstable volatile compounds that are rapidly oxidized (Anet 1969), which may explain why (*E*,*E*)-α-farnesene emission did not differ between dually infested Col-0 plants at low or high aphid density.

Phytohormonal crosstalk between JA- and SA-mediated signalling pathways is thought to underlie plant-mediated interactions with multiple insect species and behavioural responses of parasitoids and predators (Zhang et al. 2013; Stam et al. 2014; Wei et al. 2014). Activation of SA-signalling in response to aphid feeding (Moran et al. 2002; Mewis et al. 2006; Kusnierczyk et al. 2011) may suppress JA-dependent indirect defence responses. This may result in changes in the composition of the volatile blend (Truong et al. 2014). On the other hand, we found that feeding by *P. xylostella* caterpillars alone induced not only JA-regulated terpenoid volatiles in *A. thaliana* but, similar to the finding of Zhang et al. (2013), also relatively high levels of MeSA, the methyl ester of SA. This indicates that the general pattern of negative crosstalk between SA- and JA-dependent signalling pathways in the interactions between simultaneous feeding caterpillars and aphids does not always apply. Interestingly, the data show that mutation in *BSMT1* (that catalyses the synthesis of MeSA from SA) interfered with the responsiveness of *D. semiclausum* to host-infested plants. Similarly, it was shown by Snoeren et al. (2010) that the attraction of *D. semiclausum* was negatively affected by MeSA.

Induction of linalool and 1-penten-3-ol emission depends on the JA-signalling pathway (Fisher et al. 2003; Van Schie et al. 2007; Snoeren et al. 2010), whereas emission of these plant volatiles in response to caterpillar feeding was not affected by simultaneous *B. brassicae* feeding. Since it is known that the emission pattern of HIPVs varies over time and a time lag occurs between gene induction and subsequent volatile emission (Dudareva et al. 2006; Heil 2008), effects on HIPV emission by simultaneous feeding by caterpillars and aphids might have been found at other time points after induction. For example, an increase in the amount of emitted volatiles was found after 48 h in *A. thaliana* plants simultaneously infested by *P. xylostella* caterpillars and whiteflies compared to plants infested by caterpillars alone (Zhang et al. 2013).

In line with Houshyani et al. (2013) and Zhang et al. (2013), we also observed preference of *D. semiclausum* parasitoids for volatile blends from *P. xylostella*-infested *A. thaliana* plants. Mutations in the biosynthesis of linalool (*tps10* mutants) could have modified the volatile blend after infestation by *P. xylostella* caterpillars and these changes reduced the attraction of *D. semiclausum* parasitoids to host-infested plants. Therefore, linalool may function as *D. semiclausum* attractant. Linalool has been reported before as an important attractant for *D. semiclausum* parasitoids (Houshyani et al. 2013) and has been found in *P. xylostella*-induced volatile blends from *A. thaliana* (Zhang et al. 2013; Online Resource 4). Other volatile compounds induced most strongly by feeding of *P. xylostella* caterpillars were myrcene and TMTT and, therefore, may contribute to the attraction of *D. semiclausum* parasitoids (Online Resource 4). These two specific compounds were also found in the volatile blend emitted by *A. thaliana* plants in response to *P. xylostella* infestation after 2 days of feeding (Zhang et al. 2013).

In conclusion, we have shown that the behavioural response of parasitoids to HIPVs emitted by plants dually attacked by aphids and caterpillars depends on aphid density and found changes in the HIPV blend associated with density. Biosynthesis and emission of (*E*,*E*)-α-farnesene were linked to the observed preference of *D. semiclausum* parasitoids for volatiles emitted by plants dually infested by caterpillars and aphids at a high density. In addition, biosynthesis of linalool and (*E*,*E*)-α-farnesene strongly influenced *D. semiclausum* responsiveness to host-infested plants. Parasitoids are enemies of herbivorous insects that are important members of plant-associated communities. Here, we have investigated the effects of non-host herbivory on foraging behaviour of naïve parasitoids to assess effects early in parasitoid life. Parasitoids are well known to associatively learn environmental cues (Turlings et al. 1993; Hoedjes et al. 2011). Thus, they may also learn to associate cues related to encounters with hosts in multi-herbivore infestations. First evidence indicates that foraging by the parasitoid *Cotesia glomerata* is indeed influenced by previous oviposition experiences on plants infested by not only their hosts but also non-host herbivores (de Rijk 2016). As plants growing under field conditions are commonly attacked by multiple insect herbivores at the same time, a better understanding of how plants regulate indirect defence mechanisms in response to multiple insect attack and how parasitoids deal with plant-produced information will provide important knowledge on plant-mediated ecological interactions within plant-associated communities. Such multi-species interactions may be complex in themselves. Yet, the fact that non-host herbivore densities modulate these species interactions presents an additional layer of complexity.

## Electronic supplementary material

Below is the link to the electronic supplementary material.

Supplementary material 1 (PDF 218 kb)

Supplementary material 2 (PDF 290 kb)

Supplementary material 3 (PDF 209 kb)

Supplementary material 4 (PDF 342 kb)

Supplementary material 5 (PDF 302 kb)

Supplementary material 6 (PDF 283 kb)

Supplementary material 7 (PDF 284 kb)

Supplementary material 8 (PDF 293 kb)
