# Supplementary material for: Terpenoid biosynthesis in Arabidopsis attacked by caterpillars and aphids: effects of aphid density on the attraction of a caterpillar parasitoid
Source: Oecologia. 2017 Oct 20;185(4):699–712. doi: 10.1007/s00442-017-3985-2 (PMC5681606; doi:10.1007/s00442-017-3985-2)
Supplement: Supplementary file 1 — Supplementary material 1 (PDF 218 kb) [file 442_2017_3985_MOESM1_ESM.pdf]

Terpenoid biosynthesis in *Arabidopsis* attacked by caterpillars and aphids: effects of aphid density on the attraction of a caterpillar parasitoid

Anneke Kroes
Berhane T. Weldegergis
Francesco Cappai
Marcel Dicke*
Joop J.A. van Loon

Laboratory of Entomology, Wageningen University, P.O. Box 16, 6700 AA Wageningen, The Netherlands

* Corresponding author: Marcel Dicke (marcel.dicke@wur.nl)

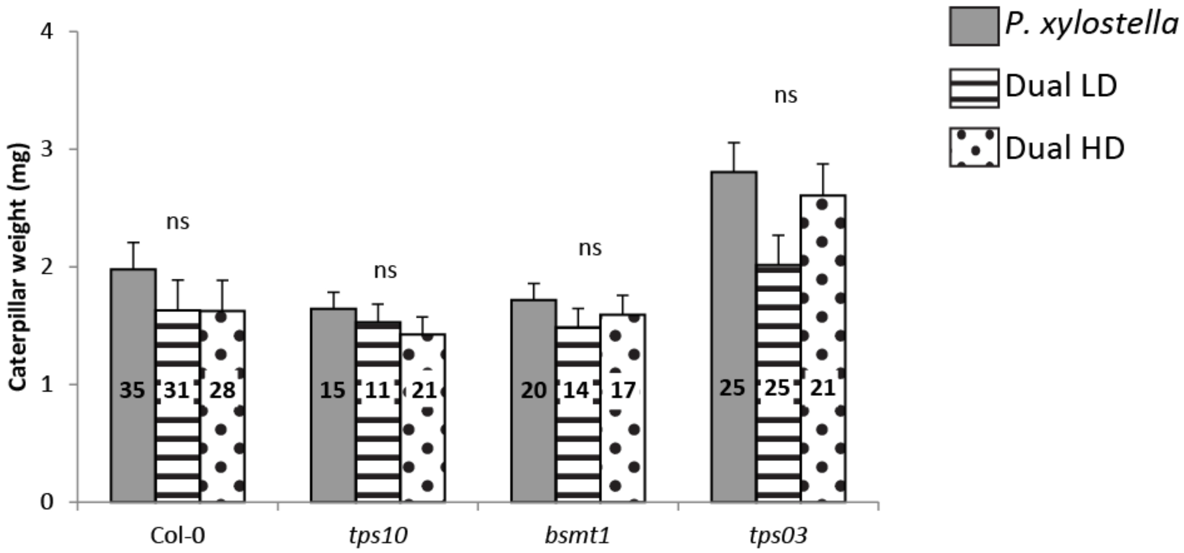

Supplemental material 1. Body mass of *P. xylostella* caterpillars after feeding during three days on *A. thaliana* wild-type Col-0 plants and mutants *tps10*, *bsmt1* and *tps03* used during the Y-tube olfactometer bioassays after single *P. xylostella* or dual *P. xylostella* and *B. brassicae* infestation (Dual). Plants were infested with either a low (LD, 5 aphids) or high density (HD, 25 aphids) of aphids and two second-instar caterpillars. For each treatment a set of four plants was used. Numbers inside each bar represent the total number of caterpillars weighed. Bars represent means ± SE (Linear Mixed Model; ns, not significant)
