# Supplementary material for: Terpenoid biosynthesis in Arabidopsis attacked by caterpillars and aphids: effects of aphid density on the attraction of a caterpillar parasitoid
Source: Oecologia. 2017 Oct 20;185(4):699–712. doi: 10.1007/s00442-017-3985-2 (PMC5681606; doi:10.1007/s00442-017-3985-2)
Supplement: Supplementary file 3 — Supplementary material 3 (PDF 209 kb) [file 442_2017_3985_MOESM3_ESM.pdf]

Terpenoid biosynthesis in *Arabidopsis* attacked by caterpillars and aphids: effects of aphid density on the attraction of a caterpillar parasitoid

Anneke Kroes
Berhane T. Weldegergis
Francesco Cappai
Marcel Dicke[*]
Joop J.A. van Loon

Laboratory of Entomology, Wageningen University, P.O. Box 16, 6700 AA Wageningen, The Netherlands

[*] Corresponding author: Marcel Dicke (marcel.dicke@wur.nl)

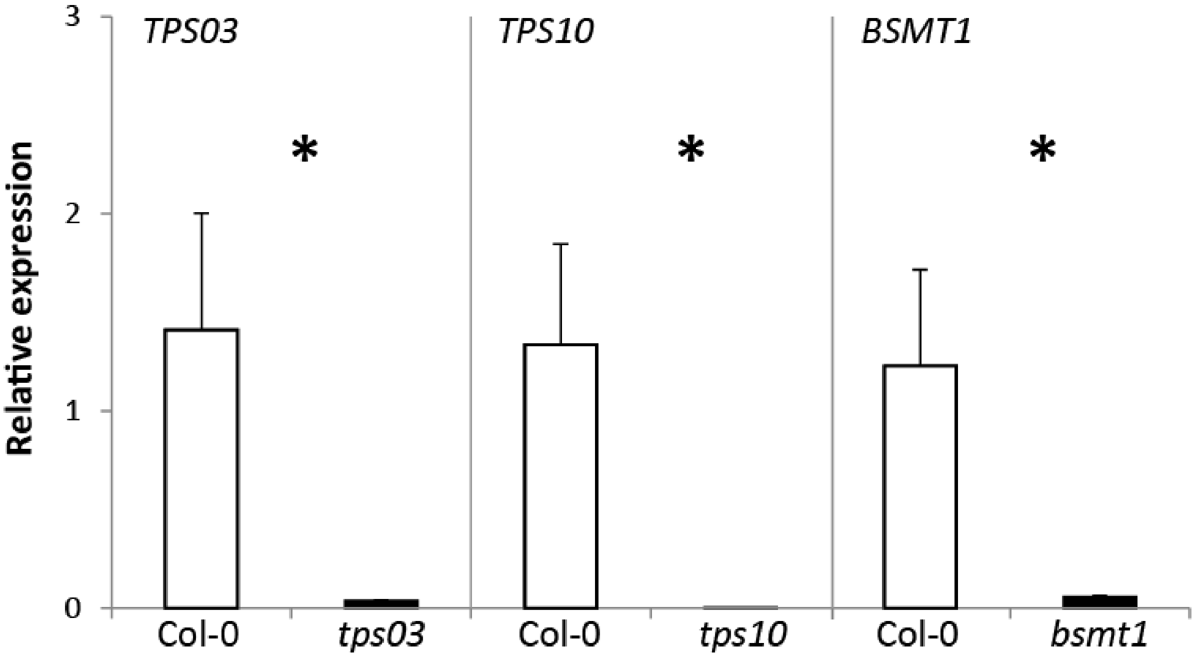

Supplemental material 3. Verification of *TPS03*, *TPS10* and *BSMT1* relative expression in leaves of *A. thaliana* wild-type Col-0 and mutants *tps03*, *tps10* and *bsmt1* after single *P. xylostella* infestation. Bars represent means ± SE (n = 4 biological replications). Asterisk, P < 0.02 (GLM)
