# Supplementary material for: Terpenoid biosynthesis in Arabidopsis attacked by caterpillars and aphids: effects of aphid density on the attraction of a caterpillar parasitoid
Source: Oecologia. 2017 Oct 20;185(4):699–712. doi: 10.1007/s00442-017-3985-2 (PMC5681606; doi:10.1007/s00442-017-3985-2)
Supplement: Supplementary file 4 — Supplementary material 4 (PDF 342 kb) [file 442_2017_3985_MOESM4_ESM.pdf]

Terpenoid biosynthesis in *Arabidopsis* attacked by caterpillars and aphids: effects of aphid density on the attraction of a caterpillar parasitoid

Anneke Kroes
Berhane T. Weldegergis
Francesco Cappai
Marcel Dicke*
Joop J.A. van Loon

Laboratory of Entomology, Wageningen University, P.O. Box 16, 6700 AA Wageningen, The Netherlands

* Corresponding author: Marcel Dicke (marcel.dicke@wur.nl)

Supplemental material 4. Volatile organic compounds emitted by *Arabidopsis* wild-type Col-0 after single *P. xylostella* infestation, dual *P. xylostella* and *B. brassicae* infestation and without infestation (Undamaged). Dual infestation was done with either a low (LD) or high (HD) *B. brassicae* density. Plants were sampled at 72 h after insect infestation.

ID	Compound	[A]Quantifier (target) ion (m/z)	[B]LRI_{Exp}	[C]LRI_{Lit}	Treatment (n = 7)			
					Undamaged	*P. xylostella*	Dual LD	Dual HD
					[D]Peak area/g plant fresh weight ± SE			
	**Alcohols**							
1	1-Penten-3-ol	57	661	672	568 ± 191[a]	1873 ± 334[b]	1567 ± 186[b]	2092 ± 345[b]
14	6-Methyl-1-octanol	97	1145	1145[E]	93 ± 25	251 ± 79	242 ± 105	248 ± 126
35	12-Methyl-(*E,E*)-2,13-octadecadien-1-ol	121	1928	NF	122 ± 27	142 ± 35	133 ± 31	148 ± 36
36	2-Methyl-(*Z,Z*)-3,13-octadecadien-1-ol	121	1943	NF	155 ± 35	168 ± 27	163 ± 40	183 ± 46
39	2-Methyl-(*E,E*)-3,13-octadecadien-1-ol	121	1968	NF	86 ± 17	102 ± 25	98 ± 22	105 ± 23
	**Terpenoids**							
3	β-Citronellene	67	947	944[E]	78 ± 11	100 ± 27	92 ± 29	121 ± 39
4	Camphene	93	952	952	12 ± 1	14 ± 2	17 ± 2	16 ± 3
5	Cyclohexene, 4-ethenyl-1,4-dimethyl-	93	960	949[F]	61 ± 12	79 ± 17	81 ± 17	87 ± 21
6	Myrcene	93	992	992	131 ± 14	211 ± 45	171 ± 21	165 ± 28
7	Limonene	136	1032	1033	591 ± 84	722 ± 135	742 ± 123	755 ± 171
8	1,8-Cineole	139	1034	1034	10 ± 5	8 ± 1	20 ± 12	10 ± 2

9	Sylvestrene	93	1039	1032	44 ± 17	69 ± 42	23 ± 5	25 ± 4
10	(*E*)-β-Ocimene	93	1049	1050	63 ± 11	89 ± 21	72 ± 12	73 ± 15
11	Y-Terpinene	93	1062	1062	245 ± 40	245 ± 49	301 ± 58	287 ± 66
12	Terpinolene	93	1092	1092	44 ± 7	74 ± 19	61 ± 17	80 ± 34
13	Linalool	93	1101	1101	59 ± 14	153 ± 48	91 ± 27	92 ± 27
15	Menthone	154	1160	1160	54 ± 38	33 ± 11	97 ± 74	35 ± 12
16	Isomenthone	154	1171	1170	13 ± 9 (n=5)	8 ± 2	21 ± 15(n=6)	10 ± 3
17	Menthol	123	1179	1177[E]	293 ± 177	264 ± 95	527 ± 395	257 ± 128
18	α-Terpineol	121	1197	1198	60 ± 15	97 ± 25	59 ± 12	63 ± 11
22	Limonene, 1,2,8,9-diepoxy-	81	1376	NF	136 ± 34	196 ± 56	203 ± 41	199 ± 51
24	Isodauca-6,9-diene	161	1387	NF	9 ± 1	11 ± 1	11 ± 1	10 ± 1
25	Longifolene	161	1423	1423	21 ± 2	25 ± 3	24 ± 3	25 ± 3
26	α-Cedrene	119	1429	1430	28 ± 2	33 ± 4	30 ± 3	35 ± 5
27	(*E*)-β-Caryophyllene	133	1434	1434	9 ± 2 (n=5)	7 ± 1 (n=6)	9 ± 1 (n=5)	8 ± 1
28	(*E*,*E*)-α-Farnesene	93	1512	1512	34 ± 4[a]	124 ± 33[b]	94 ± 16[b]	104 ± 19[b]
29	(*E*,*E*)-TMTT[*]	81	1582	1589[E]	95 ± 31[a]	791 ± 302[b]	640 ± 165[b]	513 ± 158[b]
	**Esters**							
19	Methyl salicylate	152	1201	1201	15 ± 2[a]	153 ± 73[b]	81 ± 20[ab]	67 ± 21[ab]
20	Linalyl acetate	93	1257	1257	151 ± 53	202 ± 62	134 ± 48	155 ± 64
21	α-Terpinyl acetate	93	1355	1347[E]	44 ± 8	58 ± 11	53 ± 13	46 ± 10
23	Neryl acetate	136	1384	1386	7 ± 1 (n=6)	10 ± 3	8 ± 2	9 ± 3 (n=6)
30	Methyl jasmonate, *cis*	153	1661	1654[E]	239 ± 47	193 ± 49	221 ± 66	218 ± 37
41	Nerolidol isobutyrate	121	1999	NF	187 ± 40	222 ± 57	209 ± 47	235 ± 53
	**Others**							
2	Allyl isothiocyanate	99	886	887	267 ± 143 (n=5)	347 ± 186 (n=4)	24 ± 11 (n=5)	124 ± 46
34	Farnesyl acetaldehyde	136	1845	1856[E]	55 ± 12	44 ± 12	68 ± 19	47 ± 13
	**Unknowns**							
31	unknown compound	164	1754	NA	289 ± 76	358 ± 109	356 ± 96	391 ± 107
32	unknown compound	164	1764	NA	231 ± 60	288 ± 81	285 ± 74	310 ± 84

33	unknown compound	95	1787	NA	351 ± 93	433 ± 120	433 ± 117	478 ± 131
37	unknown compound	121	1957	NA	82 ± 17	98 ± 28	98 ± 22	103 ± 26
38	unknown compound	121	1961	NA	122 ± 27	137 ± 37	128 ± 30	140 ± 34
40	unknown compound	121	1987	NA	171 ± 36	198 ± 50	194 ± 42	211 ± 50

[A]: Quantifier ion used for relative quantification of the respective volatile compounds.

[B] **:** $LRI_{Exp.}$: Linear retention indices experimentally obtained on a ZB-5MSi analytical column as described in Marques et al. (2000).

[C] : $LRI_{Lit.}$: Linear retention indices obtained from NIST 2008, on a column with (5 %-Phenyl)-methylpolysiloxane stationary phase or equivalent unless stated.

[D]: Volatile emissions are given as peak area (mean ± SE) per gram plant shoot fresh weight divided by 10^5 with the number of replicates between brackets. If emission of a volatile compound was not found in all the samples of a treatment, volatile emission values are followed by the number of samples in which the compound was detected. Mean values indicated with different letters are significantly different between means (GLM, $P < 0.05$).

[E]: $LRI_{Lit.}$ obtained from Abu-Dahab et al. (2014), Adams (1995), Babushok et al. (2011) and Kos et al. (2013).

[F] : $LRI_{Lit.}$: Linear retention indices on a column with 100% methylpolysiloxane (PDMS) stationary phase or equivalent.

NA: Not Applicable

NF: $LRI_{Lit.}$ Not Found

(*E*, *E*)–TMTT: (*E*, *E*)–4,8,12-trimethyltrideca-1,3,7,11-tetraene

Abu-Dahab R, Kasabri V, Afifi FU (2014) Evaluation of the volatile oil composition and antiproliferative activity of *Laurus nobilis* L. (Lauraceae) on breast cancer cell line Models. Records of Natural Products 8: 136-147.

Adams RP (1995) Identification of essential oil components by gas chromatography/mass spectrometry, 4th edn. Allurum Publishing Corporation, Carol Stream, IL.

Babushok, VI, Linstrom PJ, Zenkevich IG (2011) Retention indices for frequently reported compounds of plant essential oils. Journal of Physical and Chemicall Reference Data 40: 043101.

Kos M, Houshyani B, Overeem A, Bouwmeester HJ, Weldegergis BT, van Loon JJA, Dicke M, Vet LEM (2013) Genetic engineering of plant volatile terpenoids: effects on a herbivore, a predator and a parasitoid. Pest Management Science 69: 302–311.

Marques FA, McElfresh JS, Millar JG (2000) Kováts retention indexes of monounsaturated C_{12}, C_{14}, and C_{16} alcohols, acetates and aldehydes commonly found in lepidopteran pheromone blends.Journal of the Brazilian Chemical Society 11: 592–599.
