# Supplementary material for: Terpenoid biosynthesis in Arabidopsis attacked by caterpillars and aphids: effects of aphid density on the attraction of a caterpillar parasitoid
Source: Oecologia. 2017 Oct 20;185(4):699–712. doi: 10.1007/s00442-017-3985-2 (PMC5681606; doi:10.1007/s00442-017-3985-2)
Supplement: Supplementary file 5 — Supplementary material 5 (PDF 302 kb) [file 442_2017_3985_MOESM5_ESM.pdf]

Terpenoid biosynthesis in *Arabidopsis* attacked by caterpillars and aphids: effects of aphid density on the attraction of a caterpillar parasitoid

Anneke Kroes
Berhane T. Weldegergis
Francesco Cappai
Marcel Dicke*
Joop J.A. van Loon

Laboratory of Entomology, Wageningen University, P.O. Box 16, 6700 AA Wageningen, The Netherlands

* Corresponding author: Marcel Dicke (marcel.dicke@wur.nl)

Supplemental material 5. Most influential volatile compounds based on their Variable Importance in the Projection (VIP > 1) value between treatments of undamaged plants versus plants infested by *P. xylostella* alone and plants infested by both *P. xylostella* and a low *B. brassicae* density (Dual LD, 5 aphids) versus plants infested by both *P. xylostella* and a high *B. brassicae* density (Dual HD, 25 aphids)

		Most influential volatile compounds		
		VIP > 1		
ID	Compound	All treatments	Undamaged vs *P. xylostella*	Dual LD vs Dual HD
	**Alcohols**			
1	1-Penten-3-ol	2.67	2.31	2.65
14	6-Methyl-1-octanol		1.41	
	**Terpenoids**			
6	β-Myrcene	1.25	1.22	
9	Sylvestrene			1.42
10	(*E*)-β-Ocimene		1.02	1.51
12	Terpinolene		1.17	1.12
13	Linalool	1.32	1.67	1.88
16	Isomenthone			1.13
18	α-Terpineol			1.77
22	Limonene, 1,2,8,9-diepoxy-	1.09		
24	Isodauca-6,9-diene		1.27	
25	Longifolene	1.54	1.47	1.75
26	α-Cedrene			1.51
28	(*E*,*E*)-α-Farnesene	2.48	2.15	1.99
29	(*E*,*E*)-TMTT*	2.60	2.33	
	**Esters**			
19	Methyl salicylate	2.33	2.15	
23	Neryl acetate		1.18	1.02
	**Others**			
2	Allyl isothiocyanate			1.28
34	Farnesyl acetaldehyde			1.42

* (*E*,*E*)-TMTT, (*E*,*E*)-4,8,12-trimethyltrideca-1,3,7,11-tetraene
