# Supplementary material for: Terpenoid biosynthesis in Arabidopsis attacked by caterpillars and aphids: effects of aphid density on the attraction of a caterpillar parasitoid
Source: Oecologia. 2017 Oct 20;185(4):699–712. doi: 10.1007/s00442-017-3985-2 (PMC5681606; doi:10.1007/s00442-017-3985-2)
Supplement: Supplementary file 7 — Supplementary material 7 (PDF 284 kb) [file 442_2017_3985_MOESM7_ESM.pdf]

Terpenoid biosynthesis in *Arabidopsis* attacked by caterpillars and aphids: effects of aphid density on the attraction of a caterpillar parasitoid

Anneke Kroes
Berhane T. Weldegergis
Francesco Cappai
Marcel Dicke*
Joop J.A. van Loon

Laboratory of Entomology, Wageningen University, P.O. Box 16, 6700 AA Wageningen, The Netherlands

* Corresponding author: Marcel Dicke (marcel.dicke@wur.nl)

Supplemental material 7: Statistical analysis of proportion of non-responsive wasps to volatiles emitted by *Arabidopsis* wild-type Col-0 and mutants *tps10*, *bsmt1* and *tps03* three days after single *Plutella xylostella* infestation, dual *Plutella xylostella* and a low (LD, 5 aphids) or high (HD, 25 aphids) *Brevicoryne brassicae* density infestation and without infestation (undamaged). Generalized Linear Model deviance table for effect of genotype and treatment combination (*e.g*. undamaged versus *P. xylostella* and Dual LD versus Dual HD). Bold numbers indicate significant effects ($P < 0.05$)

	Factor				Interaction	
	Genotype (1)		Treatment combination (2)		1 x 2	
	d.f. = 3		d.f. = 1		d.f. = 3	
	deviance	P	Deviance	P	devianc e	P
% non-responsive wasps	263.45	**<0.001**	1.30	0.53	10.84	0.36
