# Supplementary material for: Terpenoid biosynthesis in Arabidopsis attacked by caterpillars and aphids: effects of aphid density on the attraction of a caterpillar parasitoid
Source: Oecologia. 2017 Oct 20;185(4):699–712. doi: 10.1007/s00442-017-3985-2 (PMC5681606; doi:10.1007/s00442-017-3985-2)
Supplement: Supplementary file 8 — Supplementary material 8 (PDF 293 kb) [file 442_2017_3985_MOESM8_ESM.pdf]

Terpenoid biosynthesis in *Arabidopsis* attacked by caterpillars and aphids: effects of aphid density on the attraction of a caterpillar parasitoid

Anneke Kroes
Berhane T. Weldegergis
Francesco Cappai
Marcel Dicke*
Joop J.A. van Loon

Laboratory of Entomology, Wageningen University, P.O. Box 16, 6700 AA Wageningen, The Netherlands

* Corresponding author: Marcel Dicke (marcel.dicke@wur.nl)

Supplemental material 8: Statistical analysis of gene expression in leaves of *A. thaliana* wild-type Col-0 and mutants *tps10*, *bsmt1* and *tps03* at 3 d after single *Plutella xylostella* infestation, dual infestation by *Plutella xylostella* and *Brevicoryne brassicae* aphids at a low or high density and without infestation. Generalized Linear Model deviance table for effect of infestation treatment. Bold numbers indicate significant effects P < 0.05

			Factor	
			Treatment	
			df = 3	
Plant	Product	Gene	deviance	P
Col-0	(*E,E*)-α-farnesene	*TPS03*	94.98	**0.002**
	(*E,E*)-TMTT	*TPS04*	45.45	0.068
	Linalool	*TPS10*	32.48	**0.022**
	MeSA	*BMST1*	450.86	0.114
tps10	(*E,E*)-α-farnesene	*TPS03*	24.79	**0.003**
	(*E,E*)-TMTT	*TPS04*	3.97	0.473
	MeSA	*BMST1*	418.74	**0.020**
bsmt1	(*E,E*)-α-farnesene	*TPS03*	4.10	0.716
	(*E,E*)-TMTT	*TPS04*	6.45	0.313
	Linalool	*TPS10*	30.17	**0.027**
tps03	(*E,E*)-TMTT	*TPS04*	28.32	**0.010**
	Linalool	*TPS10*	68.05	**0.005**
	MeSA	*BMST1*	199.858	**0.006**
